# Cross-cultural adaptation of the Participation and Environment Measure for Children and Youth (PEM-CY) into German: a qualitative study in three countries

**DOI:** 10.1186/s12887-020-02343-y

**Published:** 2020-10-24

**Authors:** Beate Krieger, Christina Schulze, Jillian Boyd, Ruth Amann, Barbara Piškur, Anna Beurskens, Rachel Teplicky, Albine Moser

**Affiliations:** 1 School of Health Professionals, Zurich University of Applied Sciences, Winterthur, Switzerland; 2 Department of Family Medicine, Maastricht University, Maastricht, The Netherlands; 3 Dalhousie University, Halifax, Canada; 4 University Hospital Graz, Graz, Austria; 5 Research Centre for Autonomy and Participation for People with Chronic Illness, Zuyd University of Applied Sciences, Heerlen, The Netherlands; 6 CanChild, McMaster University, Hamilton, Canada

**Keywords:** Assessment, Outcome, Children, Youth, Qualitative research, Community, Social participation, Environment, PEM-CY

## Abstract

**Background:**

Concepts such as participation and environment may differ across cultures. Consequently, to use a measure like the Participation and Environment Measure for Children and Youth (PEM-CY) in other than the original English-speaking contexts, cultural adaptation needs to be assured. The aim of this study was to cross-culturally translate and adapt the PEM-CY into German as it is used in Germany, Austria and Switzerland.

**Methods:**

Fifteen parents of children and adolescents with disabilities from three German speaking countries participated in three rounds of think-aloud interviews. We followed the procedure of cultural equivalence guidelines including two additional steps. Data was analyzed by content analysis using semantic, idiomatic, experiential and conceptual equivalence.

**Results:**

Results show adaptations mainly focused on experiential and conceptual equivalence, with conceptual equivalence being the most challenging to reach. Examples of experiential equivalence included adapting the examples of activities in the PEM-CY to reflect those typical in German speaking countries. Conceptual equivalence mainly addressed aspects of “involvement” and “environment” of children and adolescents and was reached through adaptations such as enhanced instructions and structures, and additional definitions.

**Conclusions:**

This study presents a cross-cultural translation and adaptation process to develop a German version of the PEM-CY that is suitable for Germany, Austria and Switzerland. Using a modified cultural adaptation process, a culturally adapted version of PEM-CY (German) is now available for research, practice and further validation.

## Background

Concepts such as *“participation”* and *“the environment”* may differ across cultures [1]. Yet, when the World Health Organization (WHO) included these terms in the International Classification of Function, Disability and Health (ICF), the intention was to address these complex health phenomena universally for adults, children and youth [2, 3]. Participation defined as *‘involvement in life situations’* (WHO, 2001) is an important outcome for rehabilitation in adults and youth [4–6]. Environment defined as, *“the physical, social, and attitudinal environment in which people live and conduct their lives*” [2, 3] has gained importance as a factor that can be targeted by rehabilitation interventions [7, 8]. Within the global use of ICF concepts, different cultures and societies shape how “participation” and “environment” are experienced concretely in real life. As such, related assessments should be used universally [9] and at the same time reflect cultural values. However, many commonly used assessments are developed in English speaking regions such as North America or Australia [10, 11] and might not be culturally suitable in other countries. These differences have to be looked at for each region and language separately. For example, participating in football may not have the same meaning in North America as in Europe and these types of differences should be reflected in internationally used instruments.

Culture has been defined as the “total shared, learned behavior of a society or a subgroup” [12] (p22). Direct translation of an assessment without proper cultural adaptation may leave garnered data unfit for proper interpretation [13]. Specifically for participation assessments, a lack of cultural equivalence in the translated measure has been reported [14]. As conceptualization of participation may vary across cultures, the translation of assessments needs to be combined with cross-cultural adaptation [15, 16]. In addition, language can have a significant influence on thoughts [17] and it is therefore important to gain insight into end-user’s understanding when filling in an internationally translated and culturally adapted assessment.

The Participation and Environment Measure – Children and Youth (PEM-CY) [18] is one of few assessments that combines the measurement of participation and environment for children and youth. It is a standardized parent-reported assessment to determine the extent and pattern of participation of children and youth aged 5–17 years, their levels of participation, involvement, related environmental barriers and supports, and parental wishes for changes in participation. Informed by results of qualitative interviews with parents [19], the PEM-CY was tested primarily with 576 parents of children with and without disability from Canada and the USA [18] and was found to have moderate to good reliability and validity. The PEM-CY has been used to measure participation of children with specific diagnoses, such as unilateral cerebral palsy [20] and autism spectrum disorder [21], to compare participation of children with different disabilities [22] and between different settings such as home, school and community [23]. The PEM-CY has been translated and culturally adapted into a number of languages, including Korean [16], Chinese [24], Icelandic [21], Hindi (Roopa Srinivasan, personal communication), Dutch (Eftje Kern, personal communication), and Flemish (Mareike Coussens, personal communications).

A German translation of the PEM-CY was developed in Austria [25]. After investigating reliability and validity of this translated version, the author concluded that it should be culturally adapted to better fit the Austrian context (“specifically school and leisure activities” (page 155)) and to improve the comprehension of items, as this might have been the reason for low reliability rates [25]. In German speaking Switzerland [26], a revision of the format was suggested to improve “ease of comprehension and improved cultural applicability” (page 67). Thus, problems with reliability, face validity and cultural applicability in two German speaking countries have been identified [26].

To the best of our knowledge, other than the PEM-CY, no other measurement is available in German to assess child and youth participation and their respective environments. German is mainly spoken in Central Europe and is the native language to almost 100 million people. It is the official or co-official language in Germany, Austria, Switzerland, Lichtenstein, and in the German speaking communities in South Tyrol (Italy), Belgium, Luxembourg and Poland. However, different regions use different dialects and words or phrases used in one country are not understood or differ in meaning in another one. This was the main barrier to using the Austrian Version of PEM-CY [25] with German speaking parents in Switzerland [26]. As health professionals in these countries work closely together and worker mobility is high, common assessments are needed. Thus, the rational for this study is that a valid cross-cultural adaptation of the PEM-CY into German is needed, taking into account that the questionnaire should be adapted and understandable for all German-speaking regions.

The aim of this study was to cross-culturally translate and adapt the PEM-CY into German in a way that culturally represents the PEM-CY constructs of “participation” and “environment” in Switzerland, Germany and Austria. We formulated the following research question: *What changes are needed to reach cross-cultural equivalence of the PEM-CY in German as spoken in Germany, Austria and Switzerland?*

## Methods

### Design

Cross-cultural adaptation “encompasses a process which looks at both language (translation) and cultural adaptation issues in the process of preparing a questionnaire for use in another setting” (page 3) [27]. To translate and adapt the PEM-CY into German, we applied international standards and established guidelines [28] which aim “to produce equivalency between source and target, based on content” (page 3186). This six-step process includes forward and back translations, synthesis by an inter-professional expert team and pilot testing with parents [28]. To enhance the process, we added: ongoing discussion with developers (represented by the PEM-CY team from CanChild) and a final back translation. The expert committee consisted of: occupational therapists from Germany, Switzerland and Austria with experience in childhood disabilities (BK, CS, RA, JB), health care methodologists (BP, AM, AB), a professional linguist and two English native speaking non-health professionals. A translation agreement was signed between CanChild/McMaster University and the expert committee.

### Setting

This study took place within the state-supported health care and federally organized educational systems in Germany, Austria and Switzerland. All three countries aim to include children with special needs in mainstream schools, as recommended by the Salamanca declaration of UNESCO [29]. Specialized teachers and assistants provide support in the classrooms as required. Children attend Kindergarten before entering school at age 6 or 7, followed by an apprenticeship at age 15. However, due to the different national political systems, there are slight differences in regulations influencing school and health services.

### Participants and ethics

**Fig. 1:**
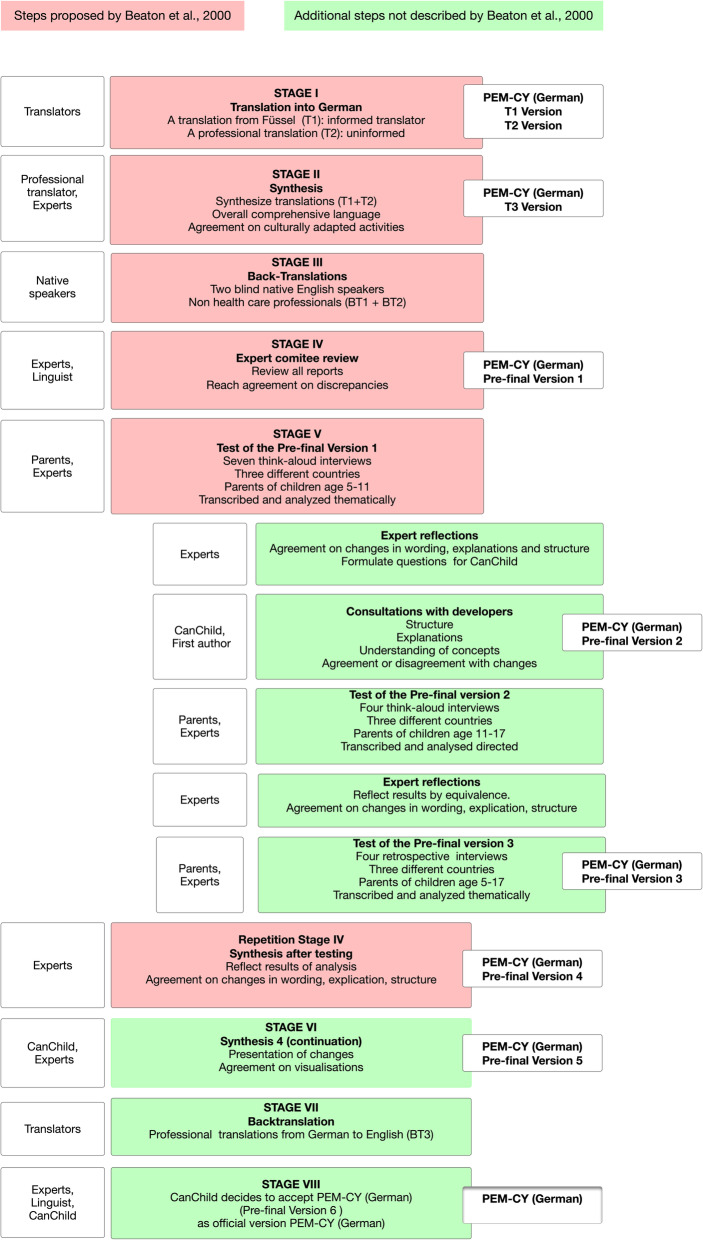
Process of cross-cultural adaptation of PEM-CY to PEM-CY (German)

Fifteen parents of children with disabilities participated in this study. Criteria for inclusion were: (1) being responsible for a child between 5 and 17 years of age with a disability, (2) living in Germany, Austria or Switzerland in the last 5 years, and (3) able to read and speak German. Parents were recruited from the work settings of the participating experts. A purposeful sampling technique was used to reach diversity. Sampling was conducted in three rounds (see Fig. 1 below). Participating parents were provided with detailed information on the study in both written and verbal formats, and they voluntarily consented to their own participation and also on behalf of their children whose data was collected, but was not the focus of analysis. Table 1 describes the characteristics of the sample.

**Table 1:** List of participants of three rounds of cognitive interviews

***Interview***		***Characteristics of parents***				***Characteristics of children***			
	**Round**	**Parenting** **role**	**Age**	**Education**	**Profession**	**Age**	**Gender**	**Medical diagnosis**	**School**
***Germany***	1	mother	34	middle	medical assistant	8	female	Trisomy 21	mainstream
	1	mother	40	middle	nurse	11	male	Visual impairment	mainstream
	1	mother	35	middle	OT	7	female	Sigmatism swallow disorder	mainstream
	2	mother	34	low	florist	12	male	ADHD	mainstream
***Austria***	1	grand-mother	57	middle	retired	6	male	ADHD	mainstream
	1	mother	45	high	economist	7	male	DCD	mainstream
	2	mother	45	middle	at home cleaner	16	male	CP	special school
	3	mother	46	high	tax adviser	6	female	Fanconi anaemia	special kindergarten
	3	mother	42	high	university assistant	8	female	Mukopolysacceri- dosis	special school
***Switzerland***	1	mother	37	middle	secretary	9	male	DCD	mainstream
	1	mother	35	middle	administrator	8	female	CP	mainstream
	2	father	40	middle	caregiver	13	male	ASD	mainstream
	2	mother	45	middle	secretary	17	female	Arthrogryphosis	intership and special school
	3	mother	54	high	economist	15	male	ASD	boarding school
	3	father mother	31 35	low low	mechanic cleaner	5	male	ASD	mainstream kindergarten

### Procedures (delineated in Fig. 1)

#### Stage I: forward translation

Following the guidelines [28], we started with two forward translations of the English PEM-CY into German. One of the translations (T1) was the existing translation conducted by an informed health scientist in Austria [25] and the second translation (T2) was prepared by a professional translator not familiar with the PEM-CY. During this stage, the cultural adaptation process focused on idiomatic and semantic equivalence.

#### Stage II: synthesis 1

The expert committee compared these two translations to develop one synthesized version (T3). The focus was on ensuring comprehension of the wording in all three countries. Activities that required cultural adaptation were identified and agreed upon.

#### Stage III: back-translation

To “*avoid information bias and elicit unexpected meaning”* [28] (p 3188) two individuals with native English, translated the synthesized version (T3) back into English (BT1 and BT2). Both translators were blinded to the original PEM-CY version, had no medical background and were fluent in German.

#### Stage IV: expert committee review

The expert committee compared BT1 and BT2 with the original PEM-CY to identify and discuss differences between the two back-translations. We considered English synonyms (such as “caring for household pets” versus “looking after pets”), differences in wording with different meaning in German (such as “community”) and differences that occurred due to cultural adaptations of activities (such as “baseball” versus “soccer” ). All unresolved differences were discussed with the PEM-CY team from CanChild who provided advice on the specific discrepancies. Next, a linguist looked at comprehension for all three German speaking countries and provided feedback on T3. All of this feedback lead to the Pre-final Version 1.

#### Stage V: tests of the pre-final versions

Pre-final Version 1 was tested with concurrent think-aloud interviews [30, 31] using a 3-step procedure [32] with seven parents of children between the age of 5–11 years. The first step included observation and listening to their thoughts, the second step involved probing and the third step focused on debriefing and recommended changes. This enabled us to assess unique higher-level thinking processes, while identifying individual differences in task performance [33]. An interview guide and personal briefing of interviewers assured a similar process in all three countries. Once themes identifying the need for changes emerged recurrently, we stopped the first round of interviews and the expert team started the analysis (see section below for details). Parent performance and suggestions for the improvements were the focus of reflection and analysis. Recommended changes were then discussed and agreed upon with CanChild.

Pre-final Version 2 was then used in a second round of interviews with parents of adolescents. Following the analysis, expert review and consultation with CanChild, we developed Pre-final Version 3 and used it to conduct retrospective cognitive interviews, in which four parents filled in the questionnaire while being observed without interruptions. The parents were then interviewed about problems, moments of hesitation and comments in general. This process was used as we expected less need for changes at this stage. Further discussion and analysis of the data led to a consensus, resulting in the PEM-CY (German) (Pre-final Version 4).

#### Stage VI: submission of the PEM-CY (German) proposal

The expert team discussed the Pre-final Version 4 with the PEM-CY team from CanChild. Although some of the proposed structural changes were not accepted by the CanChild team, other improvements led to the PEM-CY (German) Pre-final Version 5. The CanChild team requested an additional back translation to fully review and approve all changes.

#### Stage VII: back translation

A back translation of PEM-CY (German) Pre-final Version 5 was performed by a professional translator (BT3).

#### Stage VIII: final agreement

All changes were discussed with team from CanChild. The linguist checked the text and made revisions (Pre-final Version 6), leading to the final version of the PEM-CY (German).

### Analysis

The data for this study included verbatim transcriptions of the cognitive interviews, interview observation notes and minutes of all experts’ meetings. We used a directed content analysis [34], applying a-priori set of codes to search for common patterns in the data. This was guided by four types of equivalence [28]. Semantic equivalence (1) refers to the use of words that mean the same. For example, we analyzed how parents understood and explained words such as “participation” or “involvement”. Idiomatic equivalence (2) includes colloquialisms and idioms, such as “hanging out”, that may lack or have a different meaning across cultures and therefore require re-phrasing. (3) Experiential equivalence seeks to replace items with those capturing the same life experiences in another culture. This included inserting culturally relevant activities, proposed by the experts. Finally, (4) conceptual equivalence refers to concepts that linguistically cover the same logic, meaning or mental images associated with a word or phrase when transferred to another culture. We analyzed for example, how parents understood and interpreted the concepts such as “home” or the general structure of the questions used in the PEM-CY. After the first author coded changes in all four types of equivalence, part of the expert team repeated some of the analysis. Subsequently, all experts discussed the judgements consecutively, until consensus was reached on changes made to each version (see Table 2).

**Table 2:** Development of equivalence (version 1–6)

***Stage***		***Original***	***Equivalence***				***Changes made and cultural adaptations***
	Version PEM-CY	PEM-CY (English)	semantic	ideomatic	experiential	conceptual	PEM-CY (G) (German)
Stage I	Translation (T1/T2)	• gender		x			• removal of gender nomenclating for child (her or she)
		• wording (e.g. “environment”)	x				• use of different words (synonyms) e.g. environment (G: “Umfeld”, “Umwelt”)
		• wording (e.g. “community”, “involvement”)	x				• lack of direct translation into German e.g. “community” (G:“gesellschaftlich” “gemeinschaftlich”) involvement (“beteiligt” versus “engagiert”)
		• wording (e.g. school lunch)		x			• country specific wordings e.g. “Jause”(Austria) versus “Vesper”(Germany), “Znüni”(Switzerland)
Stage II	Synthesis (T3)	• wording (e.g. assignments, lunch preparation)		x			• agreement to choose words commonly used in all three countries e.g. (G: “Hausaufgaben”, “Essen zubereiten”)
		• “involvement”	x				• agreement on “being part and involved” (G. “beteiligt und eingebunden sein”)
		• activities			x		• removal of activities that are not typical in German speaking countries (e.g. “public speaking”) • addition of cultural adapted activities, e.g. “soccer”, “learning vocabulary”
		• structure				x	• present environment questions 8 (9) on next page
Stage IV	Pre-final 1	• general wording	x				• small changes in used language structure, e.g. change from survey to “questionnaire” (G: “Fragebogen”)
		• response item	x				• revised “not an issue” (G: “nicht relevant”)
**Following first round of interviews (#1 - #7)**							
Stage IV	Pre-final 2	• introduction				x	• adapt introduction - add age range of PEM-CY (5–17 years) - explain “participation” and “environment” - add a sentence that that this survey is not about the child’s independence - express that activities are just examples
		• explanations			x		• add encouragement for parents to fill in school setting
		• sequences			x		• change sequence of activity groups, start with “ Indoor play and games”
		• response items			x	x	• for school setting, add “on school days” • repeat the 4 month timeframe of frequency on each page • insert a 2 phase response option to all environmental parts: “No, it is no issue”, “yes it is an issue” and then explain supportiveness.
		• “demands”				x	• insert “demands and expectations” to questions 4–6 in environments
		• “involvement”					• change of German word for “involvement” (G: “Engagement”) (see stage II)
		• structure				x	• add subtitles to A (frequency), B (involvement), C (wished changes) • add line for comments (voluntary) for all activity groups
**Following second round of interviews (#8 - #11)**							
Stage V	Pre-final 3	• “child” • “neighborhoods”	x				• use whenever possible “child and adolescent” • replaced by “visits to public areas”
		• explanations				x	• present a definition of home, school and community on each page
		• response items environment				x	• inserted a note that parents should think broadly about the environment sections • change the answer format: headline (A and B answers) and subtheme (B1, B2, B3) referring to hindering and supportive environments
**Following third round of interviews (#12 -#15)**							
Stage IV	Pre-final 4	• “school”				x	• add “school and kindergarten”
		• visualization				x	• visually structure the item “wished changes” with a thicker line • highlight “skip to C”
Stage V	Pre-final 5	• explanations			x		• explaining that garden or yard is part of home • explaining that way to school is part of school
		• “demands”				x	• remove “expectations” (see stage IV)
		• structure				x	• remove proposed structure (see Stage V) and verbalize supporting and hindering environment (see Fig. 2)

The Pre-final Version 6, after the backtranslation and grammar check by linguist, became the final version (see Table 3)

### Trustworthiness

Experts from three German speaking countries were involved in all phases and ensured researcher triangulation [35, 36]. Four kinds of data (translations, expert knowledge, observations and transcripts) ensured data triangulation. In the last round of interviews, participants tested the final version and provided feedback on it. To ensure transferability, we provided a thick description of the research process and data collection, including quotes from the interviews.

## Results

Results focused on the research question, “*What changes are needed to reach cross-cultural equivalence of the PEM-CY in German as spoken in the three countries?”*, are presented in two tables. Table 2 presents the type of changes made during the pre-final versions 1 to 6, which are organized and explained according to the four type of equivalences in Table 3. We illustrate each type of equivalence with quotes taken from the transcribed interviews and examples from field observations made while parents completed the Pre-final Versions 1–3 of PEM-CY (German).

**Table 3:** Synthesis of needed equivalence changes to develop the PEM-CY (German)

***Equivalent changes***	***Explanation***	***Original PEM-CY***	***Changes made to culturally adapt the PEM-CY (German)***
*Semantic equivalence*	A similar semantic expression	• “community”	• “societal” (G: “gesellschaftlich”)
		• “involvement”	• “engagement” (G: “Engagement”)
		• “participation”	• “participation” G: “Teilhabe”
		• “children”	• “children and adolescents” • (G: “Kinder und Jugendliche ”)
*Idiomatic equivalence*	Less misleading expression	• “the child”- he or she	• neutral form (it) (G: “es”)
		• “assignment” and “homework”	• “homework and assignments” (G: “Hausaufgaben”)
		• “climate”	• “seasonal conditions” (G: “Jahreszeiten”)
*Experiential equivalence*	Words that represent culturally based experiences	• activity examples used in the original PEM-CY that are not common for children in the targeted countries: “Brownies”, “mentoring”, “lunchroom supervisor”, “public speaking”, “working in a store”, “baseball”	• removed uncommon examples and added new examples: “playing Lego©”, “showering”, “learning vocabulary”, “going to school”, “bullying and misuse”, “soccer”, “skiing”, “walking”, “salary during apprenticeship and internship”.
		• “Video gaming” is the first group of activities in the home setting	• “Video gaming” moved and is now the third group of activities in in the home setting
		• school participation: response option “daily”	• school participation – response option changed to “daily at school days”
		• school participation	• kindergarten and school participation
		• “neighborhood”	• “visits in public area”
*Conceptual equivalence*	Covering linguistically the same logic, meaning or mental images		**Additional information**: • enhanced definition of “participation” and “environment” • added information about the possible age of children (5–17 years) • explained that it is not about independence • included the time frame of four months on each page • added encouragement to fill in school setting despite little knowledge • instructed global thinking in the “environmental sections” • provided examples for all environmental items **Structural changes**: • add headings for all participation aspects (frequency, involvement, desire for change) • insert “activity group” as subheading • make thicker lines between options for wishes for changes • highlight “Skip to C” • explain environment and settings on each page • repetition of “environmental factor” in possible answers • present all questions with the same answer format in the environment part on the same page. • added line for possible comments for desired changes in participation

### Semantic equivalence

Overall, the four words “participation”, “involvement”, “community” and “children” could not be simply translated and needed in-depth discussion between the linguists and the expert team. These words were tested and discussed with end-users during the cognitive interviews. Given the importance of each of these words to the PEM-CY, the resulting changes are discussed below.

For “participation”, two German words exist. An equivalent of “to take part” (G: “teilnehmen”) and a more complex, less common word “to participate” (G: “teilhaben”), used mainly in professional language. Parents often read “teilnehmen” where “teilhaben” was written. Despite expressing unfamiliarity with the word “teilhaben”, they preferred it for its’ deeper meaning. Therefore, “teilhaben” is used in the adapted PEM-CY (German).

One mother mentioned a problem when judging her child’s participation and the inherent cultural norms related to inclusive programs:

*“ ...should I judge whether she takes part with healthy children or with other children with disabilities? I have to confess that my daughter hardly participates in settings with healthy children. Either she is accompanied by us, or she participates in activities that are geared for children with disabilities. Like swimming. If she is expected to participate in swimming here in the town, regularly, the answers would be completely different.” (P #13)*

The word *“involvement”* presented a different challenge. It is a multifaceted concept for which no single German translation exists. One mother explained some difficulties in judging her child’s involvement:

*“The word “independence” would have fit better to our situation. Yes, he can do everything, he just has to be reminded. Sometimes, he goes shopping for me or picks up apples at a farm [...] How involved he is? I don’t know. He makes it for me, but if he would not be obliged to do it, he would do something else. But when he does it, he is reliable, everything is perfect, he can handle the money and he behaves decently. For involvement I consider his joy and his motivation. He may not have it, but nevertheless, he performs well.” (P #2).*

This mother added that observing independence is easier than observing involvement. Most parents mentioned motivation in connection with involvement. Some spoke about an inner process of being present and active. Others connected it with being interested, curious and open. One mother mentioned that involvement also depends on others.

*“It means he takes part actively, but he also has to be accepted by others.” (P #5)*

These aspects could not be transferred directly into one German word. After the second round of interviews, we consulted with the linguist and agreed upon “engagement” (German (G): “Engagement”) and “being engaged” (G: “engagiert sein”).

Revisions for the two additional words, “community” and “children”, were more straightforward. As there was no direct translation for “community”, we selected “societal” (German “gesellschaftlich”) as the best alternative to refer to any public space or social entity outside the home and school. Finally, the PEM-CY directions uses the word “children” to apply to ages 5 to 17 years. In German, children over the age of 14 are called “adolescents” (G: “Jugendliche”). Similar to the “Y” for “youth” in the title PEM-CY, “adolescents” needed to be mentioned throughout the measure.

### Idiomatic equivalence

Three changes could be interpreted as belonging to this category. First, in the English version, “child” is always referred to as he/she. As “child” in German is neutral (“das Kind”), we did not need to use gender differences. Secondly, the expert committee agreed to use one single word (G: “Hausaufgaben”) for all school-tasks done at home (E: “homework” and “assignments”). Thirdly, some parents were confused by the word “climate” when translated into German, and its’ relevance at school.

*“Weather condition, climate? <laughs> …This does not make sense. Climate at school? I don’t question this. I presume that there is enough light and enough heating and no rain in the classroom.”* (P. #6)

We therefore chose the wording “seasonal conditions” (G: “Jahreszeiten”) to align with the original meaning of outdoor climate.

### Experiential equivalence

To capture the different cultural experiences, several examples for activities from the original PEM-CY were removed from the German version. Examples of these include “mentoring”, acting as “lunchroom supervisor”, “public speaking”, “working in a store”, being a “Brownie”, and playing “baseball”. Although these activities are known in German speaking contexts, they do not have the same cultural importance to serve as examples in the PEM-CY (German). The experts agreed on more typical cultural activities such as “playing Lego®”, “showering”, “learning vocabulary”, “going to school”, “bullying and misuse”, playing “soccer”, “skiing”, “walking” and receiving “salary during apprenticeship and internship”. Parents did not raise any concerns with the proposed activities. However, some were surprised that “computer and video games” was the first activity mentioned in the home setting.

*“My child is 6 years of age. I am proud that video gaming is an activity he did not yet discover. But I am rather astonished to see it here as the first listed activity.” (P. #5)*

To address parent concerns, “computer and video games” was moved to the third item in the home setting in the PEM-CY (German).

In the school setting, parents rarely chose the option “daily” for participation frequency because their children were not attending school on weekends. We therefore changed it from “daily” to “daily on school days”. As school starts in German-speaking countries generally at 6 or 7 years of age, we added the word “kindergarten” in the school section, to include 5 year-old children.

In the community setting, a mother indicated that her daughter with arthrogryposis never participates in neighborhood outings like shopping or going to a movie.

*“If you skip the word “neighborhood”, I would answer this question totally different. My daughter is 17 years, she uses public mobility and accesses with her scooter, malls and cinemas in the city. “Neighborhood” is even not our village, it is narrower, just here, our neighbors.” (P #13)*

As such, the term ‘neighborhood’ was considered too restrictive to best reflect the equivalent idea in Germany, Austria and Switzerland. We replaced it with the term ‘’in public’ (G: “in der Öffentlichkeit”), to be sure parents understood this to include public spaces that are beyond the immediate vicinity of the home, but within reach of public transport, for example.

### Conceptual equivalence

To ensure conceptual equivalence of the PEM-CY (German), the expert group used three different kinds of adaptations: adding additional information, improving the visual structure and providing opportunities for parents to add comments.

Additional information was needed in various forms: first, definitions of “participation” and “environment” were added in the introduction, as parents who did not understand English missed this information and aim from the title – PEM-CY. This was also helpful as parents interpreted these concepts in many different ways, with one misinterpretation shown in the following example:

*“Participation for me is when my child does something with somebody else, thus not necessarily alone. Therefore, I have problems with the participation item “computer games”, because here he is alone. At least two persons must be involved to talk about “participation”.” (P #8).*

Next, adding the age range of the assessment helped parents to understand that not all activities are suited to their child. For example, young children would not be expected to use technology for socializing. Also, as parents in the first round often mixed up “independence” and “participation”, we strongly emphasized that this assessment is not about independence. Further, the settings (home, school, and community) are explained on each page because parents were confused about separating these settings clearly. For example, parents wondered if the yard of the house was part of the home setting or already part of the community setting. Finally, parents often forgot the time frame of four months during scoring. Mentioning this on each scoring page of participation supported a more correct answering pattern.

The school setting was another area in which additional information was needed. Contrary to the home setting where parents saw themselves as a knowledgeable informant, most hesitated to fill in the school setting section. When children don’t talk about school experiences, parents feel uninformed about school. It involved also cultural aspects, as one mother reasoned:

*“I have the time to support my child at school. But this is not wished for. I could attend and bring him to activities at school. So, I could choose “yes”. Theoretically. But at the entrance of the school is a sign indicating “from here on I can do it all by myself”. We parents should not enter the school.” (P #6)*

Other parents mentioned that everything is okay as long as teachers don’t complain. Therefore, an additional sentence in the PEM-CY (German) encourages parents to fill the school part out according to their knowledge gained from conversations with their child and/or teachers.

**Fig. 2:**
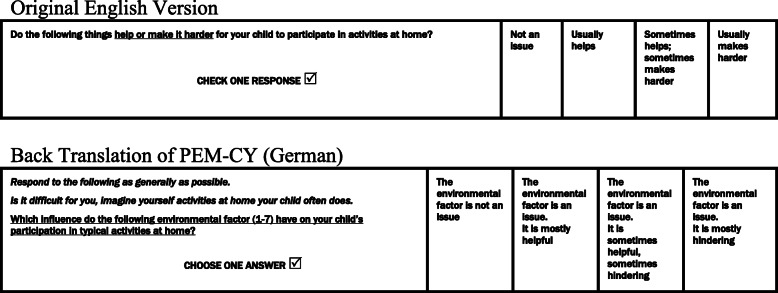
Comparison of changes from PEM-CY to PEM-CY (G) in the Environment Section

When considering the environment section, we found it supportive to add examples to all environmental questions. In the first and second round of interviews, parents had difficulties with the change of focus from the detailed participation part to the general environment part. We provided additional instructions to help them think more globally (see Fig. 2). To further support their understanding, each item of environment was explained using examples of activities. While the original PEM-CY asks about “the physical demands of typical activities in the home (e.g., strength, endurance, coordination)”, this was changed in the German version to “the physical demands for activities at home, referring to the strength, endurance and coordination required (e.g., during playing, dressing, cooking)”. Generally, the three items that ask about “physical, cognitive and social-communicative demands” were difficult for most participants. The mother of a girl with arthrogryposis explained:

*“When I think of emptying the dishwasher[...] it is clear that it is harder for her than for her brother. But I do not expect her to do the same as her brother. So, what are here demands? I don’t understand[...] is this a requirement or demands I judge and pose on her? Of course, she removes the plastic items and not the heavy loads. I don’t know what to do here. This does not make sense to me.” (P.#13)*

The team from CanChild indicated that their intention was to focus on activity demands (and not expectations of others), leading to a rephrasing in German to emphasize different activities.

To improve the visual structure of the questionnaire and make it easier to complete, titles were added to the three scoring aspects of participation (frequency, involvement and change desired). This change was needed because in the first round, parents confused these aspects or did not fill them out separately. Titles made the three categories more explicit and it was easier to refer to them in the introduction. Parents also had difficulties understanding that the “activities” are just examples. We titled these activities as “activity groups” and instructed them in the introduction to choose one or two activities out of an activity group. This supported understanding. Finally, as proposed by parents, we inserted thicker lines between the different types of change desired. This was intended to help parents to stay in the correct row and mark the correct answer.

In the first round of interviews, parents also had difficulties comprehending the concept and logic based on the format of most questions in the environment section. A mother of a child with DCD for example stated:

*“I don’t understand this theme “environment” quite right. I don’t see a connection between activities, these questions and the provided answers. I can’t answer them.” (P #10)*

Parents felt overwhelmed being asked if the environmental element is (1) an issue and if it is, (2) is it available and/or adequate and whether it is (3) supporting or hindering the child’s participation. The following example illustrates how a father of a boy with autism spectrum disorder searched for an answer for the item: “The social demands of typical activities in the home (e.g., communication, interacting with others).” He read the question twice loudly and then expressed his thoughts:

*“Well, here I am asked about influence. But influence does not support. I have to make a cognitive triangle here. The scale does not fit to the question, well, I guess I have to transmit the scale to demands. I imagine myself a typical situation at home at the table. My son has some difficulties with social communicative situations…what shall I choose here. Sometimes these situations make it easier, sometimes not [reads the question again]. For me this does not fit. I…I really do not get it. It always helps sometimes and makes it harder sometimes. There is never just one direction. This is somehow much too global. I stop here, maybe later I’ll understand it better.” (P.#12)*

In addition to the item format, parents struggled with the global answer that was expected. We proposed a two-phase response option to ease these difficulties: “if it is an issue, is it helpful or does it make it harder”. This was rejected by the team from CanChild with the argument that changing to a dichotomic response may affect the psychometric properties of the assessment. Instead we agreed to insert the environmental factor into each possible answer (see Fig. 2).

Although most participants found completing the PEM-CY strenuous, during the first round they wanted the opportunity to express their concrete wishes for change in their child’s participation. Some started to underline the activity in which they wished to see changes. To address this, we added a column to offer parents space to voluntarily write additional comments. This seemed to improve their comfort in answering the questions, as there were only a few comments in the following rounds.

### Parents’ suggestions for additional PEM-CY revisions

Some changes proposed by parents were beyond the cultural adaptation of the PEM-CY. For example, parents indicated that they would like to see activities in connection with sleep and restoration, and questions about planning of activities. Some proposed to make item 5 “getting together with other people” in Home participation into two separate items, one for interacting with family members and one for guests. In the environmental sections, it was suggested that insurance coverage be included, which seems specifically important for children in need of medical or technical aids and personal assistance. As these changes would have changed the nature of the measure, they were not included in the PEM-CY (German).

## Discussion

The aim of this study was to translate and cross-culturally adapt the PEM-CY into German in a way that represents the PEM-CY constructs of “participation” and “environment” in Switzerland, Germany and Austria. Based on the number of changes as well as their relative importance, results show the adaptations mainly focused on experiential and conceptual equivalence and that these changes mainly occurred following the cognitive interviews with parents. For experiential equivalence, the main change was to adapt the examples of activities suggested in the PEM-CY to those that are more typical in German speaking countries. Conceptual equivalence most significantly addressed aspects of “involvement” and “environment” of children and adolescents. This was reached through adaptations, such as enhanced instructions and format, and additional definitions.

Three aspects are important to discuss further: firstly, the process that included end-users, a diverse group of investigators and developers of the measure. Secondly, the four types of equivalent changes (semantic, idiomatic, experiential and conceptual) that were necessary to culturally represent the PEM-CY in Germany, Switzerland and Austria without changing the measure’s construct. Finally, the interrelation between the two main concepts of the PEM-CY, “participation” and “environment”.

This study followed the cross-cultural adaptation guidelines by Beaton [28] because of their procedural clarity and broad usage [16, 37, 38]. All stages in the process were found useful and were followed. In addition, we adapted the procedure in two ways: we included the developers (PEM-CY team from CanChild) throughout the process and added two new steps (Step 6 – Synthesis with the CanChild Team and Step 7 – Second back translation). Involving the team from CanChild ensured communication and agreement on conceptual equivalence, and ongoing developer input helped to maintain the measure’s constructs. As suggested in the literature [16, 37], these steps prevented problems in operationalization and in obtaining normative intercultural comparison at a later stage [14, 39]. Although pilot testing is not fully described in Beatons’ guidelines [28], we included a think-aloud method [32] with parents of children with disabilities. End-user’s perspectives are often overlooked in cross-cultural adaptations [39]. The insights about language and comprehensiveness of the PEM-CY that were gained by the think-aloud interviews lead to major equivalent changes in our study. Similar effects are reported with the cross-cultural adaptation of another participation measure, the Pediatric Evaluation of Disability Inventory - Computer Adaptive Test (PEDI-CAT), when translated and adapted into Dutch [37]. Specifically, the fact that all participants were parents of children and adolescents with disabilities, challenged our perceptions and understanding of normative participation. Feedback from parents with severely disabled youth, led us to question the norms and values that typically define participation in mainstream activities. In line with family-centered care and service-user involvement in general [40], we strongly recommend including end-users in future cross-cultural adaptation studies through both interviews and membership in expert committees.

To develop the PEM-CY (German), all four equivalences were used. Some of the changes were easy to address, particularly with our diverse study team. For example, idiomatic equivalence was easily addressed by the linguists. Experiential equivalence that focused on activity examples were handled by the expert team. These changes were expected based on similar processes [16, 41] that found activities are highly variable among different cultures [42]. Agreeing on which activities to revise for all three countries was a straightforward adaptation. This was particularly true for the school items. Although the school system varies greatly between North America and German speaking countries [43], the differences between Switzerland, Germany and Austria were minimal. Thus, a common experience could be formulated, and these changes were hardly questioned by the end-users during the pilot testing phase.

The two remaining equivalences, namely semantic equivalence and conceptual equivalence, were more difficult to separate and, in our experience, were more challenging for the team to reach an agreement. In cross-cultural adaptations of participation measures, specifically the conceptual aspects are often not addressed [14] and we found it was best to do so through several steps of pilot testing with end-users. We illustrate this issue with the term/concept of “involvement”. A missing suitable word in German (usable equally as a noun and as an adverb) was primarily a semantic issue. However, it became conceptual when parents expressed different understandings of “involvement”. This has been discussed in the participation literature [5]. Involvement implies provided and given access, engagement, and taking part actively rather than conforming to a given social norm [44]. It further includes emotional elements such as motivation, persistence, social connection and level of affect [5]. Proposed by parents, the experts decided to use the German word “Engagement”, which is in line with current scientific discourse [5, 42, 45]. Involvement also contains aspects of persistence [5] and the German counterpart “Engagement”(G) includes endurance. However, parents of children with disabilities in our study pointed to the fact that the involvement of their children during activities is highly variable. For example, children with ADHD or ASD who have executive functioning limitations, might have difficulties starting activities that they later engage in energetically. Further, as endurance might be weak for some children, parents are confronted with the question of whether they focus their judgement on time or quality. While these examples illustrate conceptual challenges, the investigators decided to assign this to “semantic” equivalence, as this was the starting point of the need for a discussion around cultural equivalence.

The environment sections of the PEM-CY also presented conceptual challenges. After filling in a detailed participation section, the generalization required by the environment questions was not well understood by parents. That is, parents filling in the PEM-CY (German) expected to rate the impact of the environment on each of the activities, rather than taking a more general view and considering the impact on participation in all activities within a particular setting (i.e., home, school or in the community). This problem reflects a theoretically known difficulty in measuring environment within an ICF framework [46]. Whiteneck and Dijkers describe three coding options: (1) environmental factors are coded alone, without relating these codes to activities and participation. Environmental factors are (2) coded for the ICF component of activity and participation generally and (3) environmental factors are coded in combination with capacity and performance qualifiers for each item of activities and participation separately [46]. Participants filling in the environmental section of PEM-CY expected the third version, as they also answered the participation sections (frequency, involvement and wishes for change) for each activity separately. For conceptual clarity, the statement *“respond to the following as generally as possible”* was added to the PEM-CY (German) in all three environmental sections. As often recommended in cultural adaptation processes, this added example helped to avoid confusion over item meaning [41].

While the cross-cultural process followed in this study had many strengths, the analysis revealed an interesting pattern. Comparison of original PEM-CY and the two backtranslations (BT1 and BT2) revealed a total of 102 differences in wording or phrasing of the questionnaire. 56% of the differences was caused by English synonyms, 22% of differences were due to English words that cannot be translated in their full meaning into German, thus reflecting aspects of semantic equivalence. 18% of differences occurred due to activity adaptations, reflecting experiential equivalence, the expert team had made in stage II. For only 4% of the differences, the reason could not be determined (e.g. “not an issue” (O) versus “not applicable”). It was not possible to determine the correspondence between the total equivalent changes and the number of differences. However, the changes made following the cognitive interviews with parents were primarily categorized as experiential equivalence, changes that were not identified by the expert team when developing the first pre-final version.

One limitation of this work is that due to the complexity of the PEM-CY, it was not possible to reach the level of comprehension of a twelve year old, as recommended in the guidelines [28]. As a result, parents with lower literacy levels needed help completing the measure. This suggests that the clinicians and researchers may need to modify the self-completed administration of the PEM-CY (German) when it is being used with parents who have reduced German language level or low literacy. This is of particular relevance as the rate of foreign population ranges between 12% in Germany and 22% in Switzerland [47]. An additional limitation is that due to our inclusion criteria, some of the results may not be relevant for parents of children without disabilities. Similar research with parents of children without disability did not find problems in answering the environmental sections [24]. It could be that parents of children with disabilities, who face more environmental barriers compared to parents of children without disabilities [23, 48, 49], display more difficulties in answering questions in the environment section. Finally, the selected guidelines [28] did not contain “operational” or “measurement” equivalence, as described in other guidelines [50] and this may limit the generalizability of the findings.

## Further research and practical implications

As with all culturally-adapted measures, the PEM-CY (German) needs to be tested to establish psychometric properties and validated in different research and clinical contexts, and with different groups of children and adolescents [28, 51]. It also has to be disseminated and its practical use in the German speaking context examined. In addition, existing measures with the same constructs (e.g. the Young Children’s Participation and Environment Measure (YC-PEM) [52] and the PEM+[53]) should be coordinated with this present adaptation to maintain consistency in wording.

Referring to the practicability of the PEM-CY, parents strongly felt it should be completed together with a therapist face-to-face to jointly identify intervention goals and strategies [54]. Filling it in independently, did not provide them with new insights per se, and some parents of children with severe health conditions felt overly disappointed as they were reminded of their childs’ limitations. If a therapist works with the family while they are completing the PEM-CY, the therapist can coach the family and enable them to see the possibilities for their child.

## Conclusions

This study presents a cross-cultural translation and adaptation process to develop a German version of the PEM-CY that is suitable for three German speaking countries. As participation and environment are both complex concepts to measure, conceptual equivalence posed the greatest challenges for this cultural adaptation. With extensive input from parents, expert therapists and researchers, as well as the PEM-CY team from CanChild, a culturally adapted version of PEM-CY (German) is now available for research, practice and further validation.

## Data Availability

The used datasets and analysis of this study are available from the corresponding author on reasonable request.

## Abbreviations

PEM-CY: The Participation and Environment Measure – Children and Youth
WHO: World Health Organization
ICF: International Classification of Functioning, Disability and Health
T1: Translation 1
T2: Translation 2
T3: Synthesis of Translation 1 and 2
O: Original Version
BT1: Backtranslation 1
BT2: Backtranslation 2
BT3: Backtranslation 3
G: German

## References

[1] World Health Organization (2011). World report on disability 2011. Am J Phys Med Rehabil.

[2] WHO. International classification of functioning, disability and health (ICF). World Health Organization; 2001. Available from: http://www.who.int/classifications/icf/en/. Cited 2015 Nov 15.

[3] WHO. International classification of functioning, disability, and health: children & youth version: ICF-CY. Geneva; 2007. Available from: https://apps.who.int/iris/bitstream/handle/10665/43737/9789241547321_eng.pdf;jsessionid=7156EA288F409320ABA80F7DA54493FB?sequence=1. Cited 2015 Nov 15.

[4] Adair B, Ullenhag A, Keen D, Granlund M, Imms C (2015). The effect of interventions aimed at improving participation outcomes for children with disabilities: a systematic review. Dev Med Child Neurol.

[5] Imms C, Granlund M, Wilson PH, Steenbergen B, Rosenbaum PL, Gordon AM (2017). Participation, both a means and an end: a conceptual analysis of processes and outcomes in childhood disability. Dev Med Child Neurol.

[6] Heinemann AW. Putting Putting outcome measurement in context: a rehabilitation psychology perspective. Rehabil Psychol. 2005;50:6–14.

[7] Anaby D, Law M, Majnemer A, Feldman D, Mercerat C, Tremblay S (2018). The effectiveness of the pathways and resources for engagement and participation (PREP) intervention: improving participation of adolescents with physical disabilities. Dev Med Child Neurol.

[8] Kramer JM, Helfrich C, Levin M, Hwang IT, Samuel PS, Carrellas A (2018). Initial evaluation of the effects of an environmental-focused problem-solving intervention for transition-age young people with developmental disabilities: Project TEAM. Dev Med Child Neurol.

[9] Simeonsson RJ, Leonardi M, Lollar D, Bjorck-Akesson E, Hollenweger J, Martinuzzi A. Applying the international classification of functioning, disability and health (ICF) to measure childhood disability. Disabil Rehabil. 2003;25:602–10. Available from: http://www.ncbi.nlm.nih.gov/pubmed/12959334. Cited 2014 Nov 12. 10.1080/0963828031000137117 12959334

[10] Adolfsson M, Malmqvist J, Pless M, Granuld M. Identifying child functioning from an ICF-CY perspective: everyday life situations explored in measures of participation. Disabil Rehabil. 2011;33:1230–44. Available from: http://www.ncbi.nlm.nih.gov/pubmed/20958202. Cited 2014 Nov 12. 10.3109/09638288.2010.526163 20958202

[11] Chien C-W, Rodger S, Copley J, Skorka K. Comparative content review of children’s participation measures using the International Classification of Functioning, Disability and Health-Children and Youth. Arch Phys Med Rehabil. 2014;95:141–52. Elsevier Ltd. Available from: http://www.ncbi.nlm.nih.gov/pubmed/23851418. Cited 2014 Oct 28. 10.1016/j.apmr.2013.06.027 23851418

[12] Mead M, Mead M, Metraux R (1953). The study of culture at a distance. Study Cult. A distance.

[13] Morrison SD, Rashidi V, Banushi VH, Barbhaiya NJ, Gashi VH, Sarnquist C (2013). Cultural adaptation of a survey to assess medical providers’ knowledge of and attitudes towards HIV/AIDS in Albania. PLoS One.

[14] Stevelink S, van Brakel MWH (2013). The cross-cultural equivalence of participation instruments: a systematic review. Disabil Rehabil.

[15] Monticone M, Baiardi P, Ferrari S, Foti C, Mugnai R, Pillastrini P (2012). Development of the Italian version of the Pain Catastrophising Scale (PCS-I): cross-cultural adaptation, factor analysis, reliability, validity and sensitivity to change. Qual Life Res.

[16] Jeong Y, Law M, Stratford P, DeMatteo C, Kim H (2016). Cross cultural validation and psychometric evaluation on the participation and environment measure of children and youth in Korea. Disabil Rehabil.

[17] Prinz J. Culture and cognitive science. Stanford Encycl Philos. 2016. Available from: https://plato.stanford.edu/archives/fall2016/entries/culture-cogsc.

[18] Coster W, Bedell G, Law M, Khetani MA, Teplicky R, Liljenquist K, et al. Psychometric evaluation of the participation and environment measure for children and youth. Dev Med Child Neurol. 2011;53:1030–7. Available from: http://www.ncbi.nlm.nih.gov/pubmed/22014322. Cited 2014 Nov 7. 10.1111/j.1469-8749.2011.04094.x 22014322

[19] Bedell GM, Khetani MA, Cousins MA, Coster WJ, Law MC. Parent perspectives to inform development of measures of children’s participation and environment. Arch Phys Med Rehabil. 2011;92:765–73. 10.1016/j.apmr.2010.12.029. Elsevier Inc. 10.1016/j.apmr.2010.12.029 21530724

[20] Mitchell LE, Ziviani J, Boyd RN (2015). Characteristics associated with physical activity among independently ambulant children and adolescents with unilateral cerebral palsy. Dev Med Child Neurol.

[21] Egilson ST, Jakobsdóttir G, Ólafsson K, Leósdóttir T. Community participation and environment of children with and without autism spectrum disorder: parent perspectives. Scand J Occup Ther. 2017;24:187–96. Informa UK Limited, trading as Taylor 8 Francis Group. Available from: http://www.tandfonline.com/doi/full/10.1080/11038128.2016.1198419. 10.1080/11038128.2016.1198419 27329683

[22] Tint A, Maughan AL, Weiss JA (2017). Community participation of youth with intellectual disability and autism spectrum disorder. J Intellect Disabil Res.

[23] Anaby D, Law M, Coster W, Bedell G, Khetani M, Avery L, et al. The mediating role of the environment in explaining participation of children and youth with and without disabilities across home, school, and community. Arch Phys Med Rehabil. 2014;95:908–17. 10.1016/j.apmr.2014.01.005. Available from: http://www.archives-pmr.org/article/S0003-9993(14)00032-X/abstract%5Cnhttp://www.embase.com/search/results?subaction=viewrecord&from=export&id=L53049424%5Cnhttp://search.ebscohost.com/login.aspx?direct=true&db=rzh. 10.1016/j.apmr.2014.01.005 24468018

[24] Chien CW, Li-Tsang CWP, Cheung PPP, Leung KY, Lin CY (2019). Development and psychometric evaluation of the Chinese version of the participation and environment measure for children and youth. Disabil Rehabil.

[25] Füssel C. Partizipation bei Kindern und Jugendlichen mit und ohne Beeinträchtigungen - ein Vergleich im Rahmen der Übersetzung und Validierung der “Participation and Environment Measures - Child and Youth (PEM-CY).” University of Vienna; 2014.

[26] Boyd J. Face validity, internal consitency and applicability of the Participation and Environment Measure: Child and Youth (German Translation) in Switerland: a mixed methods pilot study. Dalhousie Univ. 2018. Available from: https://dalspace.library.dal.ca//handle/10222/73809.

[27] Beaton DE, Bombardier C, Guillemin F, Ferraz MB. Recommendations for the cross-cultural adaptation of health status measures. New York: American Academy of Orthopeadic Surgeons 12; 2002.

[28] Beaton DE, Bombardier C, Guillemin F, Ferraz MB (2000). Guidelines for the process of cross-cultural adaptation of self-report measures. Spine (Phila. Pa. 1976).

[29] UNESCO. The Salamanca statement and framework for action on special needs education. Paris; 1994.

[30] Willis GW (2005). Cognitive interviewing - A tool for improving questionnaire design.

[31] Charters E (2003). The use of think-aloud methods in qualitative research. An introduction to think-aloud methods. Brock Educ.

[32] Hak T, van der Veer K, Jansen H (2008). The Three-Step Test-Interview (TSTI): an observation-based method for pretesting self-completion questionnaires. Surv Res Methods.

[33] Ericsson KA, Simon HA (1980). Verbal reports as data. Psychol Rev.

[34] Hsieh H-F, Shannon SE (2005). Three approaches to qualitative content analysis. Qual Health Res.

[35] Guba EG, Lincoln YS, Guba EG, Lincoln YS (1985). Establishing trustworthiness. Nat. Inq.

[36] Nowell LS, Norris JM, White DE, Moules NJ (2017). Thematic analysis: striving to meet the trustworthiness criteria. Int J Qual Methods.

[37] Bos N, Engel MF, Van Rijswijk NJ, Verheijden JMA, Coster W, Moed R (2019). Translation and cross-cultural adaptation of the PEDI-CAT: Dutch version. J Pediatr Rehabil Med.

[38] Chang R, Yang H (2019). Cross-cultural adaptation of the management of agression and violence attitude scale for nurses in emercency departments.

[39] Soto S, Linas K, Jacobstein D, Biel M, Migdal T, Anthony BJ (2015). A review of cultural adaptations of screening tools for autism spectrum disorders. Autism.

[40] Wong Chung R, Willemen A, Voorman J, Ketelaar M, Becher J, Verheijden J (2019). Managing oneself or managing together? Parents’ perspectives on chronic condition self-management in Dutch pediatric rehabilitation services. Disabil Rehabil.

[41] Arestad KE, MacPhee D, Lim CY, Khetani MA. Cultural adaptation of a pediatric functional assessment for rehabilitation outcomes research. BMC Health Serv Res. 2017;17:1–13. BMC Health Services Research. 10.1186/s12913-017-2592-6 PMC5603009 28915817

[42] Hammel J, Magasi S, Heinemann A, Whiteneck G, Bogner J, Rodriguez E (2008). What does participation mean? An insider perspective from people with disabilities. Disabil Rehabil.

[43] LeTendre GK, Hofer BK, Shimizu H. What is tracking? Cultural expectations in the United States, Germany, and Japan. Am Educ Res J. 2003;40:43–89. SAGE Publications. Available from: http://journals.sagepub.com/doi/10.3102/00028312040001043. Cited 2019 Nov 17.

[44] Coster W, Khetani MA. Measuring participation of children with disabilities: issues and challenges. Disabil Rehabil. 2008;30:639–48. Available from: http://www.ncbi.nlm.nih.gov/pubmed/17852316. Cited 2014 Sep 29. 10.1080/09638280701400375 17852316

[45] King G, Rigby P, Batorowicz B. Conceptualizing participation in context for children and youth with disabilities: an activity setting perspective. Disabil Rehabil. 2013;35:1578–85. Available from: http://www.ncbi.nlm.nih.gov/pubmed/23311673. Cited 2014 Nov 12. 10.3109/09638288.2012.748836 23311673

[46] Whiteneck G, Dijkers MP (2009). Difficult to measure constructs: conceptual and methodological issues concerning participation and environmental factors. Arch Phys Med Rehabil.

[47] OECD. Foreign population (indicator). OECD; 2019.

[48] Khetani M, Marley J, Baker M, Albrecht E, Bedell G, Coster W (2014). Validity of the Participation and Environment Measure for Children and Youth (PEM-CY) for Health Impact Assessment ( HIA ) in sustainable development projects. Disabil Health J.

[49] Lamash L, Bedell G, Josman N. Participation patterns of adolescents with autism spectrum disorder compared to their peers: parents’ perspectives. Br J Occup Ther. 2019;83(2):78–87.

[50] Herdman M, Fox-Rushby J, Badia X (1998). A model of equivalence in the cultural adaptation of HRQoL instruments: the universalist approach. Qual. Life Res..

[51] De Bock F, Bosle C, Graef C, Oepen J, Philippi H, Urschitz MS (2019). Measuring social participation in children with chronic health conditions: Validation and reference values of the child and adolescent scale of participation (CASP) in the German context. BMC Pediatr.

[52] Khetani M, Graham J, Davies P, Law M, Simeonsson R (2015). Psychometric properties of the young children’s participation and environment measure. Arch Phys Med Rehabil.

[53] Jarvis JM, Gurga A, Greif A, Lim H, Anaby D, Teplicky R, et al. Usability of the Participation and Environment Measure Plus (PEM+) for client-centered and participation-focused care planning. Am J Occup Ther. 2019;73:7304205130p1. Available from: http://www.ncbi.nlm.nih.gov/pubmed/31318677. Cited 2019 Nov 26. 10.5014/ajot.2019.032235 PMC9563087 31318677

[54] Khetani M, Cliff A, Schelly C, Daunhauer L, Anaby D. Decisional support algorithm for collaborative care planning using the Participation and Environment Measure for Children and Youth (PEM-CY): a mixed methods study. Phys Occup Ther Pediatr. 2014;1–22. Available from: http://www.ncbi.nlm.nih.gov/pubmed/24670061. Cited 2014 Nov 12. . 10.3109/01942638.2014.899288 24670061

